# Effects of *Nigella sativa* (*Habbatus sauda*) Oil and Nicotine Chronic Treatments on Sperm Parameters and Testis Histological Features of Rats

**DOI:** 10.1155/2014/218293

**Published:** 2014-05-21

**Authors:** Ng Cho Ping, Noor Hashida Hashim, Durriyyah Sharifah Hasan Adli

**Affiliations:** ^1^Institute of Graduate Studies, University of Malaya, 50603 Kuala Lumpur, Malaysia; ^2^Center for Foundation Studies in Science, University of Malaya, 50603 Kuala Lumpur, Malaysia; ^3^Institute of Biological Sciences, Faculty of Science, University of Malaya, 50603 Kuala Lumpur, Malaysia

## Abstract

Twenty-four *Sprague-Dawley* male rats (7–9 weeks old, 200–250 g) were divided into Nicotine (N) (0.5 mg/100 g body weight (BW), Nicotine Control (NC) (saline, 0.1 mL/100 g BW), *Habbatus sauda* oil (HS) (6.0 **μ**L/100 g BW), and *Habbatus sauda* Control (HSC) (corn oil, 0.1 mL/100 g BW) groups and treated for 100 days. Sperm parameters and seminiferous tubules measurements were evaluated. The N showed a significantly lower sperm motility (1.03 ± 0.05 × 10^6^ sperm/mL) and percentage of normal (82.61 ± 0.03%) and live (93.88 ± 0.01%) sperm, higher value for the seminiferous tubule (253.36 ± 1.83 **μ**m) and lumen (100.15 ± 2.38 **μ**m) diameters and spermatogonia (19.85 ± 0.39 **μ**m) and spermatocytes (33.37 ± 0.59 **μ**m) layers, and thinner spermatid-sperm layer (22.14 ± 0.71 **μ**m) than the NC (*P* < 0.05). The HS had significantly higher sperm motility (1.49 ± 0.04 × 10^6^ sperm/mL) and percentage of normal (90.61 ± 0.01%) and live (96.98 ± 0.01%) sperm, smaller lumen diameter (67.53 ± 2.34 **μ**m) and thinner spermatogonia (17.67 ± 0.32 **μ**m) and wider spermatid-sperm (36.95 ± 0.79 **μ**m) layers than the HSC (*P* < 0.05). This research confirmed that nicotine reduced sperm motility and morphology of normal and live sperms and also affected the testis histology, while *Habbatus sauda* oil increased sperm quality and gave better testis histological features.

## 1. Introduction

Infertility is a major medical problem worldwide affecting 10–15% of couples globally. Infertility is demonstrated by the inability of female to become pregnant after 12 months of unprotected intercourse [1].  Male infertility is more prevalent compared to female [2] and one of the major factors that lead to infertility in male is smoking. The major constituents of cigarette smoke that affect health are nicotine and tar in the particulate phase and carbon monoxide in the gaseous phase [3].  Nicotine is considered as one of the most toxic and detrimental substances that can be found in tobacco smoke [4]. It is an alkaloid found mainly in plants and is present in high concentrations in tobacco (*Nicotiana tabacum*). Besides being a natural insecticide [5], nicotine is pharmacologically active and has a negative impact on the reproductive system and fertility of males [6, 7].

In contrast, there are traditional practices involving the use of plants for their fertility enhancing and aphrodisiac properties [8, 9]. This is partly reflected by a report from the World Health Organization (WHO) which stated that traditional medicine is used as the primary healthcare product by approximately 70–80% of the world population [10]. There were various plants used as traditional medicine such as* Camellia sinensis*,* Carica papaya*,* Thymus vulgaris*, and* Nigella sativa*. One of these plants is* Nigella sativa* (*Habbatus sauda*), a plant from the Ranunculaceae family which grows abundantly in several Middle Eastern and Southern Mediterranean countries [11].* Habbatus sauda* is from the Arabic term Habat-ul-Sauda and is referred to as Kalonji in South Asia, besides having the English name which is Black Cumin [12].  Seeds of* Nigella sativa* are used primarily as spice, in addition to being used as traditional medicine in numerous countries [13–15]. Essential oil compounds contained in the seeds of* Nigella sativa* have also been found to contribute to its various biological activities [16]. The major and active component of* Nigella sativa* is essential oil identified as thymoquinone as has been revealed by pharmacological studies [17]. Thymoquinone possesses a potent antioxidant effect, which could protect organs from oxidative damage by free radical generating agents [18, 19]. In relation to fertility, it was reported that oral administration of* Nigella sativa* oil on rats with hypercholesterolemia increased their reproductive performance, weight of seminal vesicle, level of testosterone, sperm motility, and sperm quality [20]. Oral administration of oil extracted from seeds of* Nigella sativa* for a period of 53 days has also shown improved male rat fertility [21]. This agrees well with the report that black seeds contain alkaloids and phenols, which could stimulate the secretion of testosterone and follicle stimulating hormone (FSH) [22]. The increased levels of testosterone and FSH in testicular tissue have been shown to increase sperm concentration [23]. To date, data concerning the medicinal use of* Habbatus sauda* oil on reproductive performance and testicular dysfunction is still lacking. Hence, the aims of this research were to provide relevant data on the effects of* Habbatus sauda* oil and nicotine on sperm parameters and testis histological features of rats.

## 2. Materials and Methods

### 2.1. Animal Maintenance

Twenty-four male* Sprague-Dawley* rats (7–9 weeks) with an average weight of 200–250 g were used in this experiment. Water and food in the form of standard pellets were given* ad libitum* to the rats. Wood shavings were used as bedding, which covered the bottom of cages to absorb urine. The bedding would be changed on an average of every three days to maintain a clean environment for the rats and to reduce unnecessary infection.

### 2.2. Experimental Design

The rats were randomly divided into four groups: Nicotine (N), Nicotine Control (NC),* Habbatus sauda* (HS), and* Habbatus sauda* Control (HSC) groups. Rats in the N and NC groups were intramuscularly injected with nicotine at 0.5 mg/100 g body weight and saline at 0.1 mL/100 g body weight, respectively. Rats in the HS and HSC groups were force-fed with* Habbatus sauda* oil at 6.0 *μ*L/100 g body weight and corn oil at 0.1 mL/100 g body weight, respectively. Pure* Habbatus sauda* oil (Doğaci, Turkey) was diluted with corn oil, which acted as control for* Habbatus sauda*. Treatments were carried out for 100 days and rats were sacrificed on day 101. Abstracted epididymides were immersed in Toyoda Yokoyama Hoshi (TYH) medium, which was added with Bovine Serum Albumin (BSA) (SIGMA A7030-10 g) prior to sperm parameters evaluation. The protocols used were approved by the Institutional Animal Care and Use Committee, University of Malaya (UM IACUC), with the reference number of ISB/20/04/2012/DSHA (R).

### 2.3. Sperm Parameter Evaluation

The abstracted cauda epididymides were transferred into 10 mL of Toyoda Yokoyama Hoshi (TYH) medium added with Bovine Serum Albumin (BSA) prior to sperm motility evaluation. The sperm suspension was kept in a CO_2_ incubator (Heal Force CO_2_ incubator) with 5.0% CO_2_ at 37°C. An aliquot of 40.0 *μ*L was pipetted from the sperm suspension onto a haemocytometer slide (Improved Neubauer by Hirschmann Techcolor). The haemocytometer was then left at room temperature for 5 minutes to allow sedimentation of the sperm to the grid of the counting chamber, prior to observation under a light microscope (Olympus CX21FS1) with 20x (phase contrast) objective lens. Sperm motility assessment was performed in accordance with the method as suggested in NAFA and ESHRE-SIGA, Laboratory Manual [24]. The vitality and morphology of sperm were analyzed using eosin-nigrosin (eosin Y: MERCK, 1.15935.0025) (nigrosin: MERCK, 1.15924.0025) staining technique. An aliquot of 50.0 *μ*L sperm suspension was mixed thoroughly with 50.0 *μ*L of eosin-nigrosin stain on a clean petri dish. Next, 15.0 *μ*L of the stained sperm mixture was transferred onto a glass slide and five smears were made for each rat. The glass slides were then left to dry at room temperature before being observed under a light microscope for vitality; live cells appeared to be colourless and dead cells appeared to be red or pink in colour. Approximately 200 sperms were observed for dead and live cells and the percentage of dead and live cells was recorded. As for sperm morphology, another 200 sperms were accessed for normal and abnormal head and tail defects. Classification of sperm morphology and vitality was in accordance with the guidelines as stipulated in the WHO Laboratory Manual [25].

### 2.4. Testis Histological Features

The abstracted testes were fixed in Bouin's solution for 48 hours at room temperature. Fixed testes were trimmed transversely into three parts. The middle part was then immersed in 70% alcohol followed by immersions in a series of alcohol solutions with ascending concentrations. After a dehydration process, the tissue samples were then processed further before being sectioned using a rotary microtome. A small drop of Mayer's Albumin was placed at the centre of the glass slide and spread evenly using a cleaned finger. A drop of distilled water was then placed on the same glass slide and tissue sections were transferred onto the slide. The glass slides were dried and kept in a slide box. Haematoxylin and eosin (H&E) staining technique would stain the nucleus purple and the cytoplasm pink. The steps of the H&E staining technique included deparaffinization, hydration, haematoxylin and eosin staining, dehydration, and clearing. The features evaluated were the diameter of seminiferous tubules, diameter of lumen, and width of spermatogonia layer, spermatocytes layer, and spermatid-sperm layer.

### 2.5. Statistical Analysis

Data obtained were analyzed using the Statistical Package for Social Sciences (SPSS) programme. All percentage data were subjected to arcsine transformation before statistical analysis was done. Data were analyzed through analysis of variances (ANOVA) with significant levels of *P* < 0.05.

## 3. Results

### 3.1. Sperm Parameters

#### 3.1.1. Effects of Nicotine on Sperm Parameters

Intramuscular administration of 0.5 mg/100 g nicotine for a period of 100 days negatively affected all sperm parameters studied (Table 1). It significantly lowered sperm motility (1.03 ± 0.05 × 10^6^ sperm/mL) compared to the control group (1.31 ± 0.04 × 10^6^ sperm/mL) (*P* < 0.05). The N group had a significantly lower percentage of normal sperm (82.61 ± 0.03%) besides having a significantly higher percentage of abnormal head (3.69 ± 0.01%) and tail defect (13.50 ± 0.02%) sperm when compared to the NC group (*P* < 0.05). A significantly lower percentage of live sperm (93.88 ± 0.01%) and a significantly higher percentage of dead sperm (6.12 ± 0.01%) were recorded for rats in the N group compared to the NC group (*P* < 0.05).

#### 3.1.2. Effects of HS on Sperm Parameters

Oral administration of 6.0 *μ*L/100 g* Habbatus sauda* oil performed on rats for a period of 100 days had positive effects on all sperm parameters studied, except for the presence of sperm with abnormal head defects (Table 2). The treatment significantly increased sperm motility (1.49 ± 0.04 × 10^6^ sperm/mL) compared to the control group (1.33 ± 0.06 × 10^6^ sperm/mL) (*P* < 0.05). The percentage of normal sperm (90.61 ± 0.01%) was significantly higher while the percentage of sperm with tail defects (7.06 ± 0.01%) was lower compared to the control group (*P* < 0.05). A significantly higher percentage of live sperm was observed for the HS group (96.98 ± 0.01%), which also had a significantly lower percentage of dead sperm (3.02 ± 0.01%) compared to the HSC group (*P* < 0.05).

### 3.2. Testis Histological Features

#### 3.2.1. Testis Histological Features of Nicotine-Treated Rats

**Figure 1:**
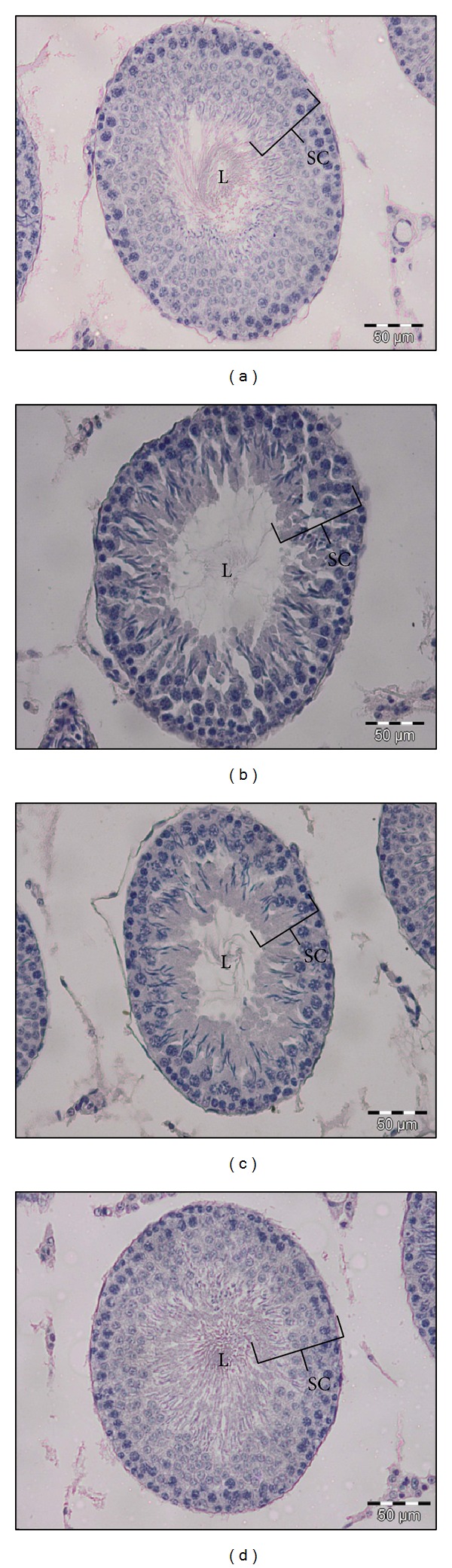
Seminiferous tubules at 200x magnification for (a) NC group, (b) N group, (c) HSC group, and (d) HS group. Note: L = lumen of seminiferous tubule and SC = spermatogenic cells.

Significantly higher values were obtained for the N group for all testis histological features studied, except for the width of spermatid-sperm layer (Figures 1(a) and 1(b) and Table 3). Bigger seminiferous tubule diameter (253.36 ± 1.83 *μ*m) and lumen diameter (100.15 ± 2.38 *μ*m) were found. Thicker spermatogonia layer (19.85 ± 0.39 *μ*m) and spermatocytes layer (33.37 ± 0.59 *μ*m) were observed, in contrast to spermatid-sperm layer (22.14 ± 0.71 *μ*m) which was significantly thinner than the NC group (*P* < 0.05).

#### 3.2.2. Testis Histological Features of* Habbatus sauda*-Treated Rats

Generally,* Habbatus sauda* treatment gave mixed results of the features studied (Figures 1(c) and 1(d) and Table 4). The HS group had a significantly smaller lumen diameter (67.53 ± 2.34 *μ*m) and thinner spermatogonia layer (17.67 ± 0.32 *μ*m), although a significantly thicker spermatid-sperm layer (36.95 ± 0.79 *μ*m) as compared to the HSC group was observed (*P* < 0.05). No significant difference was detected for both the diameter of seminiferous tubule and the thickness of the spermatocytes layer.

## 4. Discussion

Rats were chosen as the experimental animal in this research for their well-defined reproductive systems and the fact that compounds which could cause infertility in human males were also found to be active in rats [26]. The findings of the current research suggested that nicotine could cause harmful effects on the sperm quality and spermatogenic cell arrangement of male rats while* Nigella sativa* oil tended to improve it. Comparatively, this research used a lower dose of chronic nicotine treatment, while others had used higher acute nicotine treatment, for example, 20.0 mg/kg body weight (2.0 mg/100 g body weight) and 30.0 mg/kg body weight (3.0 mg/100 g body weight) of aqueous extract of* Nicotiana tabacum* on the male albino Wistar rats for 21 days [27]. The current research is similar to the one which used nicotine at a dose of 0.4 mg/100 g body weight daily for 3 months but limited to the effects on the ultrastructure of the rat testis [28].

The male* Sprague-Dawley* rats chronically treated with nicotine for a period of 100 days, as done in this research, showed a decrease in sperm motility. These findings were in line with studies, which found that sperm count and motility of human males were adversely affected by smoking behaviour [29]. Nicotine administration of 0.5 mg/kg and 1.0 mg/kg per body weight on rats daily for 4 weeks also found that sperm motility of rats was lowered compared to the control group [30]. Nicotine and cotinine also caused a negative effect on motility, spermatogenesis, epididymal sperm count, and fertilizing potential of sperm [6]. The male albino Wistar rats treated with 20.0 mg/kg body weight and 30.0 mg/kg body weight of aqueous extract of* Nicotiana tabacum* for 21 days also showed decreased sperm count and sperm motility [27].

Similarly, it was found that the normal morphology of sperm was adversely affected by smoking behaviour [29]. Sperm abnormalities observed in rats treated with nicotine might be due to low production of testosterone and the DNA-damaging effects caused by nicotine [31]. In addition, an increase in reactive oxygen species (ROS) level caused by nicotine would also lead to morphological defects on sperm, since mammalian sperms are rich in polyunsaturated fatty acids and are susceptible to attack by ROS  [32]. Present results showed that sperm vitality of rats treated with nicotine also showed negative feedback, hence agreeing with findings of a reduction in percentage of live sperm in a study carried out in a dose-dependent manner [30].

Since pure* Nigella sativa* oil was used in the present research, the findings obtained could be due to thymoquinone, which has been identified as the major and active compound of* Nigella sativa* by pharmacological studies, and/or other active compounds present in* Nigella sativa* oil [17]. Currently, there is no report on which compound of* Nigella sativa* would contribute to fertility in males; hence, the possibility of pure* Nigella sativa* oil as a complementary alternative substance to improve fertility of the males [33].

Our preliminary research using* Nigella sativa* oil at 2.0 *μ*L/100 g body weight for 60 days did not show any significant results on sperm parameters. The present research discovered that rats given an oral administration of* Habbatus sauda *oil at 6.0 *μ*L/100 g body weight for 100 days had increased sperm motility. Oral administration of* Nigella sativa* would increase sperm motility from cauda epididymis [34]. This increment on sperm motility might be due to the effects of* Nigella sativa* on oxidative phosphorylation enzymes [35]. In addition, alloxan diabetic male rats showed improvement in semen quantity and mobility after being orally administered with* Nigella sativa* at the dose of 2% of its diet for 30 days [36].

Current results obtained on sperm morphology were in agreement with the work which showed that rats given oral administration of* Nigella sativa* oil at a dose of 0.5 mL/day had reduced sperm abnormalities [20]. These findings were correlated with the antioxidant properties of thymoquinone, which is the main constituent of* Nigella sativa* oil [37]. Rats treated with* Habbatus sauda* oil in this current research also had a higher percentage of live sperm compared to the control group. Rats treated with* Nigella sativa* for a shorter period of 53 days tended to have increased sperm viability [34].

Results of testis histological features studied gave indications to the spermatogenesis process. In this research, rats treated with nicotine demonstrated less mature sperm in the seminiferous tubule. Thus, our research shared similar findings with a study which found a slight decrease in spermatogenic series and sperm count in seminiferous tubules of male* Swiss* albino mice treated with nicotine for durations of both one week and two weeks [38]. Male Wistar rats treated with varied doses of nicotine for 30 days also showed a reduction in germ cells and spermatids of their seminiferous tubules [39]. On the other hand, some temporary defects on testes histopathology of rats aged 7 weeks when exposed to nicotine were also reported [40]. In addition, male Wistar rats treated with nicotine for 90 days showed notable deterioration changes in seminiferous tubules, spermatids, and the Sertoli cells [6].

As for testis parameters of rats treated with* Habbatus sauda* oil, findings in this research were in line with a report of increased number of spermatids in* Nigella sativa*-treated rats [34]. Since thymoquinone is the major constituent found in* Nigella sativa*, it was believed to have protective effects on testicular parameters [41]. In another study, the protective effects of thymoquinone on testicular parameters had been proven [42]. In addition, coadministration of cisplatin and* Nigella sativa* oil on rats for a period of 21 days showed an evident improvement in the structure of testes [43].

Generally, nicotine treatment was known to increase lipid levels and lipid peroxidation products in serum and testis of rats [44, 45]. It was also reported that nicotine administration disrupted the components of the free radical defence system and tended to exert oxidative stress in germ cells [46, 47]. The free radicals produced would lead to cellular injury. The structure and fluidity of cell membrane would be altered when membrane phospholipids and lipid peroxidation were disintegrated, marked by the release of unsaturated fatty acid from membrane phospholipids [48, 49]. Based on results of our present research, decreases in sperm quality and testis histological features observed in nicotine-treated rats may be due to an increased oxidative degradation of phospholipids.

## 5. Conclusion

In conclusion, this research advocates that nicotine tended to reduce quality of sperm and affect the arrangement of spermatogenic cells while* Habbatus sauda* oil could enhance the quality of sperm and gave better features of testis histology.

## Figures and Tables

**Table 1:** Sperm motility, morphology, and vitality of Nicotine (N) and Nicotine Control (NC) groups.

Parameter	Motility (×10^6^ sperm/mL) (mean ± SE)	Morphology (%) (mean ± SE)			Vitality (%) (mean ± SE)	
		Normal	Head defect	Tail defect	Live	Dead
Treatment						
N (*n* = 6)	1.03 ± 0.05^a^	82.61 ± 0.03^a^	3.69 ± 0.01^b^	13.50 ± 0.02^b^	93.88 ± 0.01^a^	6.12 ± 0.01^b^
NC (*n* = 6)	1.31 ± 0.04^b^	88.04 ± 0.02^b^	1.77 ± 0.01^a^	9.93 ± 0.02^a^	95.46 ± 0.01^b^	4.54 ± 0.01^a^

^ab^Superscripts within the same column show significant difference (*P* < 0.05).

**Table 2:** Sperm motility, morphology, and vitality of *Habbatus sauda* (HS) and *Habbatus sauda* Control (HSC) groups.

Parameter	Motility (×10^6^ sperm/mL) (mean ± SE)	Morphology (%) (mean ± SE)			Vitality (%) (mean ± SE)	
		Normal	Head defect	Tail defect	Live	Dead
Treatment						
HS (*n* = 6)	1.49 ± 0.04^b^	90.61 ± 0.01^b^	2.19 ± 0.00^a^	7.06 ± 0.01^a^	96.98 ± 0.01^b^	3.02 ± 0.01^a^
HSC (*n* = 6)	1.33 ± 0.06^a^	85.15 ± 0.02^a^	2.71 ± 0.00^a^	11.98 ± 0.02^b^	92.93 ± 0.01^a^	7.07 ± 0.01^b^

^ab^Superscripts within the same column show significant difference (*P* < 0.05).

**Table 3:** Testis histological features of Nicotine (N) and Nicotine Control (NC) treated rats.

Parameter	Diameter of seminiferous tubules (*μ*m) (mean ± SE)	Diameter of lumen (µm) (mean ± SE)	Width of spermatogonia layer (*μ*m) (mean ± SE)	Width of spermatocytes layer (*μ*m) (mean ± SE)	Width of spermatid-sperm layer (*μ*m) (mean ± SE)
Treatment					
N (*n* = 6)	253.36 ± 1.83^b^	100.15 ± 2.38^b^	19.85 ± 0.39^b^	33.37 ± 0.59^b^	22.14 ± 0.71^a^
NC (*n* = 6)	242.75 ± 1.24^a^	79.64 ± 2.01^a^	18.82 ± 0.27^a^	30.95 ± 0.34^a^	25.40 ± 0.78^b^

^ab^Superscripts within the same column show significant difference (*P* < 0.05).

**Table 4:** Testis histological features of *Habbatus sauda* (HS) and *Habbatus sauda* Control (HSC) treated rats.

Parameter	Diameter of seminiferous tubules (*μ*m) (mean ± SE)	Diameter of lumen (µm) (mean ± SE)	Width of spermatogonia layer (*μ*m) (mean ± SE)	Width of spermatocytes layer (*μ*m) (mean ± SE)	Width of spermatid-sperm layer (*μ*m) (mean ± SE)
Treatment					
HS (*n* = 6)	252.15 ± 1.76^a^	67.53 ± 2.34^a^	17.67 ± 0.32^a^	34.57 ± 0.50^a^	36.95 ± 0.79^b^
HSC (*n* = 6)	255.97 ± 1.81^a^	92.22 ± 2.41^b^	19.04 ± 0.36^b^	33.66 ± 0.48^a^	27.12 ± 0.80^a^

^ab^Superscripts within the same column show significant difference (*P* < 0.05).
